# Synthesis and Structure-Activity Relationship of Some New Thiophene-Based Heterocycles as Potential Antimicrobial Agents

**DOI:** 10.3390/molecules21081036

**Published:** 2016-08-09

**Authors:** Yahia Nasser Mabkhot, Fatima Alatibi, Nahed Nasser E. El-Sayed, Nabila Abdelshafy Kheder, Salim S. Al-Showiman

**Affiliations:** 1 Department of Chemistry, College of Science, King Saud University, P.O. Box 2455, Riyadh 11451, Saudi Arabia; fatmah-alotaibi@hotmail.com (F.A.); nelsayed@ksu.edu.sa (N.N.E.E.-S.); showiman@ksu.edu.sa (S.S.A.-S.); 2 National Organization for Drug Control and Research, Agouza, Giza 35521, Egypt; 3 Department of Pharmaceutical Chemistry, Faculty of Pharmacy, King Khalid University, Abha 61441, Saudi Arabia; nabila.abdelshafy@gmail.com; 4 Department of Chemistry, Faculty of Science, Cairo University, Giza 12613, Egypt

**Keywords:** thiophene, enaminone, pyrazole, pyridine, antimicrobial activity

## Abstract

Several new pyrazole, pyridine, [1,2,4]triazolo[1,5-α]pyrimidine, benzimidazo[1,2-*a*]pyrimidine and 1,2,4-triazolo[3,4-*c*][1,2,4]triazine derivatives incorporating a thiophene moiety were synthesized from (*E*)-ethyl 5-(3-(dimethylamino)acryloyl)-4-phenyl-2-(phenylamino)thiophene-3-carboxylate (**1**). The structures of the newly synthesized compounds were confirmed by IR, ^1^H-, ^13^C-NMR, mass spectral data and elemental analysis. The antibacterial and antifungal activities of all the synthesized compounds were evaluated. The results indicated that compounds **9**, **12**, and **19** were found to be more potent than the standard drug Amphotericin B against *Aspergillus fumigates*. Additionally, compound **12** exhibited higher activity than the standard drug Amphotericin B against *Syncephalastrum racemosum*.

## 1. Introduction

The synthesis and study of thiophene derivatives have been of interest due to their facile synthesis and broad spectrum of biological and pharmacological activities. A literature survey indicated that heterocyclic compounds containing thiophene ring were found to exhibit wide range of biological activities, such as antioxidant [1], anti HIV [2], anesthetic [3], bactericidal [4], antifungal [5], anti-inflammatory [1,6], antitubercular [7], and antitumor [8,9,10] activities. Additionally, there is a thiophene ring embedded in a large number of biologically important compounds, such as vitamin H, anthelmintic Banminth [11], and insecticidal Xanthopappin A [12]. Moreover, many thiophene derivatives are documented as chromophoric components for photonic polymers [13], such as dyes [14] and as air stable semiconductors [15]. Enaminones are versatile starting materials for the synthesis of fused ring systems. These synthons were reacted with several nucleophilic and electrophilic reagents to construct new heterocyclic systems [16,17]. The aforementioned facts stimulated our interest for the synthesis of novel heterocyclic compounds containing thiophene ring utilizing enaminone **1** [18] as a key precursor to fulfill our objectives. We anticipated that the synthesized compounds would possess diverse pharmacological activities.

## 2. Results and Discussion

### 2.1. Chemistry

In continuation of our interest in the chemistry of thiophene compounds [19,20,21,22] to develop novel library of pharmaceutical targets, we started from the enaminone **1** as a precursor for the synthesis of novel heterocycles incorporating a thiophene core. The enaminone **1** was synthesized according to the procedure reported previously by our group [18].

**Scheme 1:**
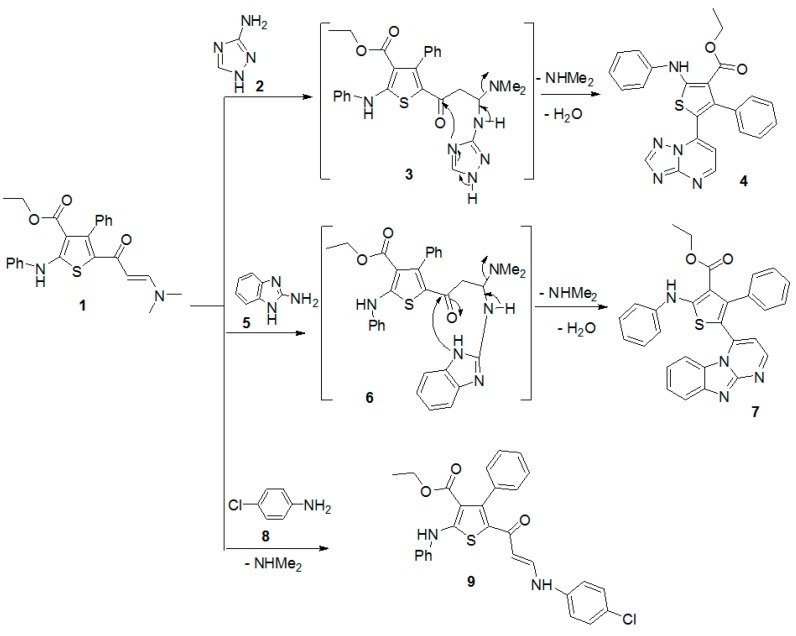
The reaction of enaminone **1** with some nitrogen nucleophiles.

Firstly, the reactivity of this enaminone with some nitrogen nucleophiles was explored. Thus, condensation of enaminone **1** with 3-amino-1*H*-[1,2,4]triazole (**2**) afforded ethyl 5-([1,2,4]triazolo[1,5-*a*]pyrimidin-7-yl)-4-phenyl-2-(phenylamino)thiophene-3-carboxylate (**4**) (Scheme 1). The structure of the latter compound was established by its spectral data and elemental analysis. For example, its IR spectrum revealed the absence of the ketonic carbonyl group present in the spectrum of the enaminone **1**. Additionally, its ^1^H-NMR spectrum revealed the disappearance of the signals corresponding to the two methyl protons of the starting enaminone **1**. In addition, its mass spectrum showed a peak at *m*/*z* 441 due to its molecular ion. It was suggested that this reaction is started by nucleophilic attack of the exocyclic amino group of the triazole to the activated double bond of the enaminone **1** to afford the Michael-type product **3**, which underwent intramolecular cyclization and the elimination of a dimethylamine and water molecules to afford the final product **4**, as outlined below in Scheme 1.

In a similar manner, the reaction of the enaminone **1** with 2-aminobenzimidazole (**5**) under the same experimental conditions gave only one isolable product, as examined by TLC. The structure of the obtained products were established as ethyl 5-(benzimidazo[1,2-*a*]pyrimidin-4-yl)-4-phenyl-2-(phenylamino)thiophene-3-carboxylate (**7**) (Scheme 1). The suggested chemical structure is in agreement with the experimental spectral data. Furthermore, the reaction of enaminone **1** with the 4-chloroaniline (**8**) gave the corresponding thiophene derivative **9** (Scheme 1). The structure of compound **9** was confirmed by its spectral and elemental data (see Experimental section). For example, its proton NMR spectrum revealed the absence of the protons of the dimethylamino group present in the spectrum of the enaminone **1**.

**Scheme 2:**
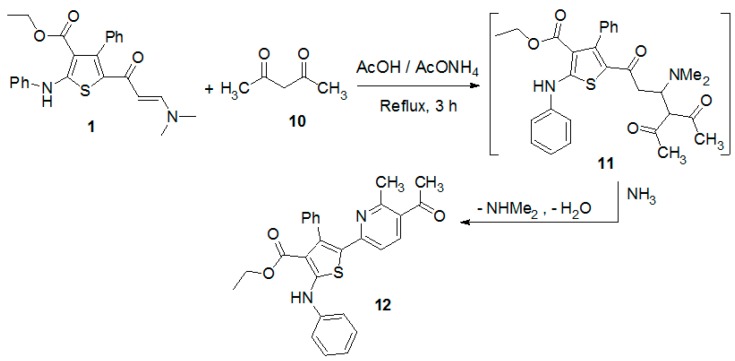
Reaction of enaminone **1** with 2,4-pentanedione.

Next, The reaction of the enaminone **1** with 2,4-pentanedione in glacial acetic acid in the presence of ammonium acetate gave the pyridine derivative **12** (Scheme 2). The structure of the product was verified by spectral data and elemental analyses (see Experimental Section).

The regioselective synthesis of new pyrazole attached to the thiophene ring system via 1,3-dipolar cycloaddition reaction of imine **13** with the enaminone **1** was also studied.

Treatment of the enaminone **1** with the imine 13 (generated in situ from 2-oxo-*N*′-phenylpropanehydrazonoyl chloride (**14**) [23,24] by the action of triethylamine in benzene) afforded a single product that was identified, on the basis of its elemental analysis and spectral (IR, ^1^H-NMR and MS) data. The structure of the isolated cycloadduct was assigned as ethyl 5-(3-acetyl-1-phenyl-1*H*-pyrazole-4-carbonyl)-4-phenyl-2-(phenylamino)thiophene-3-carboxylate (**16**) based on its elemental analyses and spectroscopic data. The ^1^H-NMR spectrum of the formed cycloadduct showed a singlet signal at δ 7.46, which corresponds to H-5 of the pyrazole ring in the product. This assignment is based on the fact that in the pyrazole ring, H-4, is more shielded than H-5. Thus, the signal of H-5 usually appears at δ 8.66–8.69 [25], whereas that of H-4 appears at δ 5.81–5.89 [26,27]. The above-mentioned information indicated that the reaction of enaminone **1** with hydrazonoyl chloride **14** is regiospecific.

**Scheme 3:**
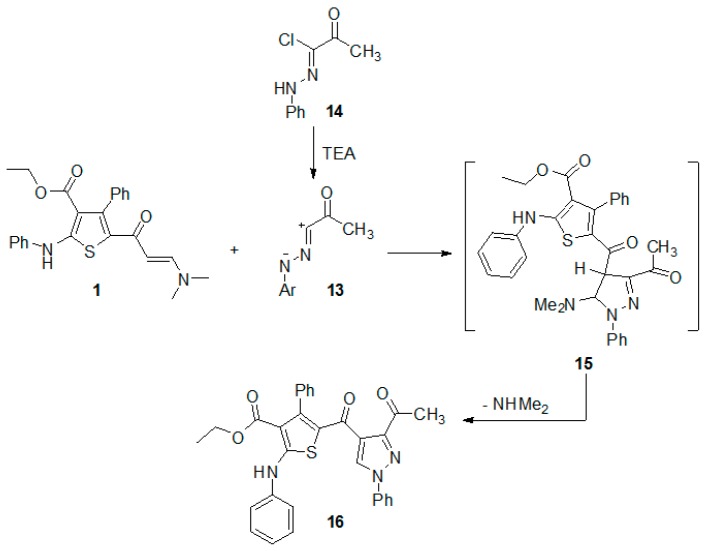
Regioselective synthesis of pyrazole derivative **16**.

To account for the formation of pyrazole **16**, we suggested that the reaction proceed via 1,3-dipolarcycloaddition of the imine **13**, derived from hydrazonoyl chloride **14**, to the activated double bond in enaminone **1** to give the respective nonisolable cycloadduct **15**, which underwent aromatization via elimination of the dimethylamine molecule to give the final pyrazole derivative **16** (Scheme 3).

**Scheme 4:**
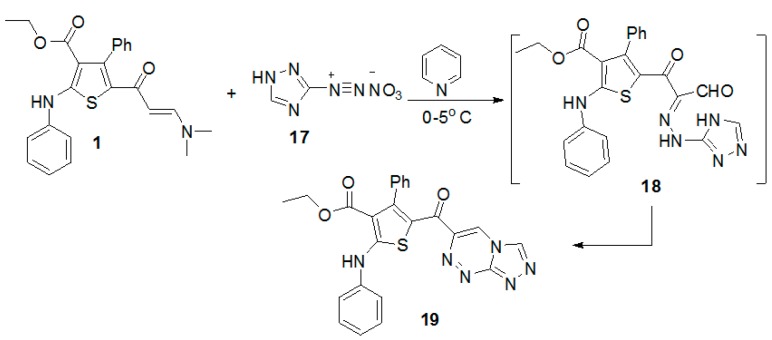
Synthesis of ethyl 5-([1,2,4]triazolo[3,4-*c*][1,2,4]triazine-6-carbonyl)-4-phenyl-2-(phenylamino)thiophene-3-carboxylate (**19**).

Finally, the coupling of enaminone **1** with the diazonium salt of 3-amino-1*H*-[1,2,4]triazole in pyridine at low temperature gave ethyl 5-([1,2,4]triazolo[3,4-*c*][1,2,4]triazine-6-carbonyl)-4-phenyl-2-(phenylamino)thiophene-3-carboxylate (**19**) (Scheme 4). The reaction proceeded by an initial formation of nonisolable intermediate **18** [28,29,30], followed by elimination of water molecule to give the final product **19**.

**Scheme 5:**
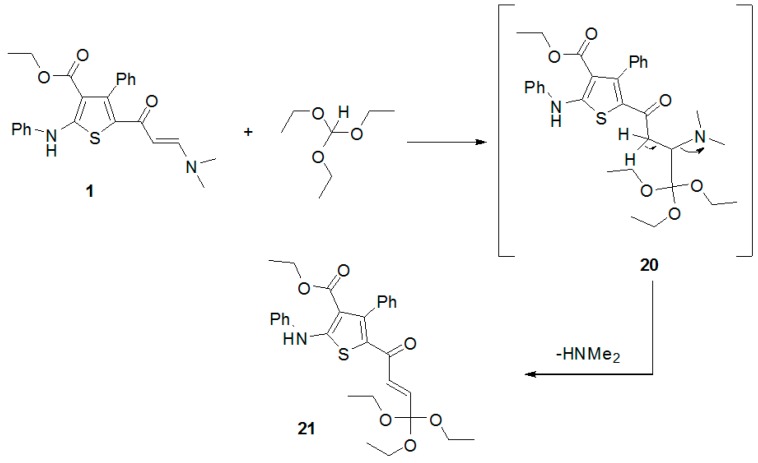
Synthesis of thiophene derivative **21**.

Refluxing enaminone **1** with triethylorthoformate gave the triethoxymethyl derivative **21** (Scheme 5). Its ^1^H-NMR spectrum showed the disappearance of the signals due to dimethylamino protons and presence of signals due to the ethoxy protons. In addition, its ^13^C-NMR spectrum revealed presence of ethoxy carbons. Moreover, its mass spectrum showed a peak at *m*/*z* 523 due to its molecular ion.

### 2.2. Antimicrobial Evaluation

The newly synthesized compounds were evaluated in vitro for their antibacterial activity against *Streptococcus pneumonia* and *Bacillis subtilis* as examples of Gram-positive bacteria, and *Pseudomonas aeruginosa* and *Escherichia coli* as examples of Gram-negative bacteria. Additionally, they were evaluated in vitro for their antifungal activity against *Aspergillus fumigates*, *Syncephalastrum racemosum*, *Geotricum candidum*, and *Candida albicans.* Ampicillin and Gentamicin were used as reference drugs for antibacterial activity, and Amphotericin B was used as reference drugs for antifungal activity [31]. These assays were conducted at the Regional Center for Mycology and Biotechnology of Al-Azhar University, Cairo, Egypt. The antimicrobial results are expressed as the mean zone of inhibition in mm ± standard deviation beyond well diameter (6 mm) produced on the microorganisms, shown in Table 1 and Table 2.

The results in Table 1 revealed that compound **16** with a pyrazole moiety revealed a high degree of antibacterial activity towards *Pseudomonas aeruginosa* and inhibition effects against *Escherichia coli.*

Data from the antifungal evaluation in Table 2 shows that compounds **9**, **12**, and **19** were found to be more potent than the standard drug, Amphotericin B, against *Aspergillus fumigates*. Additionally, compound **12** was more potent than Amphotericin B against *Syncephalastrum racemosum.* All the compounds revealed moderate activity towards *Candida albicans.*

### 2.3. Structure Activity Relationship (SAR)

By inspection of the experimental results of the antimicrobial activity of the synthesized compounds, the following structural activity relationship assumptions are suggested.

- The pyrazole moiety in compound **16** is necessary to have higher antibacterial activities towards *Pseudomonas aeruginosa*, and *Escherichia coli.*
- It is interesting to point out that 4-chlorophenylaminoacryloylmoiety in **9**, pyridine moiety in **12**, and triazolo[3,4-*c*]triazine moiety in **19** are necessary to have higher antifungal activity against *Aspergillus fumigates.*

In general, the above-mentioned results suggest that the new thiophene derivatives **9**, **12** and **19** may provide valuable leads for the synthesis and development of novel antifungal agents.

## 3. Experimental Section

### 3.1. Chemistry

#### 3.1.1. General

All the chemicals were purchased from various suppliers, including Sigma-Aldrich (St. Louis, MO, USA), and were used without further purification, unless otherwise stated. Melting points were measured on a Gallenkamp melting point apparatus (Thermo Fisher Scientific, Paisley, UK) in open glass capillaries and are uncorrected. Infrared spectra (IR) were recorded using the KBr disc technique using a Perkin Elmer FT-IR spectrophotometer 1000 (PerkinElmer, Waltham, MA, USA). ^1^H- and ^13^C-NMR spectra were recorded on either a JEOL ECP 400 NMR spectrometer (Tokyo, Japan) operating at 400 MHz or on a JEOL ECP 300 NMR spectrometer operating at 300 MHz in deuterated chloroform (CDCl_3_) or dimethyl sulphoxide (DMSO-*d*_6_). Chemical shifts are referred to in terms of ppm and *J*-coupling constants are given in Hz. Mass spectra were recorded on a Shimadzu GCMS-QP 1000 EX mass spectrometer (Tokyo, Japan) at 70 eV. Elemental analysis was carried out on a Perkin Elmer 2400 Elemental Analyzer; CHN mode. The biological evaluations of the products were carried out in the Medical Mycology Laboratory of the Regional Center for Mycology and Biotechnology of Al-Azhar University, Cairo, Egypt.

#### 3.1.2. Synthetic Procedures

##### Reaction of Enaminone **1** with Heterocyclic and Aromatic Amines

General procedure: A mixture of enaminone **1** (0.42 g, 1 mmol) and the appropriate amine derivatives **2** or **5** or **8** (1 mmol each), in absolute ethanol (20 mL) were heated under reflux for 6 h and then left to cool. The precipitated solid product was filtered off, dried and recrystallized from EtOH to afford the corresponding thiophene derivatives **4**, **7** and **9**, respectively.

*Ethyl 5-([1,2,4]triazolo[1,5-a]pyrimidin-7-yl)-4-phenyl-2-(phenylamino)thiophene-3-carboxylate* (**4**). Yield (90%), yellow powder, m.p. 230–232 °C; IR (KBr) *v* 3422 (NH), 1619 (C=O) cm^−1^; ^1^H-NMR (CDCl_3_, 300 MHz) δ 0.71 (t, 3H, CH_3_, *J* = 7.0 Hz), 3.90 (q, 2H, CH_2_, *J* = 7.0 Hz), 6.5 (d, 1H, CH, *J* = 12 Hz), 7.02–8.01 (m, 12H, ArH), 10.62 (s, 1H, NH); MS *m*/*z* (%): 442 (2.7%), 441 (M^+^, 8.5%), 440 (5.5%), 194 (99.9%). Anal Calcd. for C_24_H_19_N_5_O_2_S (441.50): C, 65.29; H, 4.34; N, 15.86. Found: C, 65.18; H, 4.22; N, 15.92%.

*Ethyl 5-(benzimidazo[1,2-a]pyrimidin-4-yl)-4-phenyl-2-(phenylamino)thiophene-3-carboxylate* (**7**). Yield (85%), yellow powder, m.p. 214–216 °C; IR (KBr) *v* 3428 (NH), 1632 (C=O) cm^−1^; ^1^H-NMR (CDCl_3_, 300 MHz) δ 0.71 (t, 3H, CH_3_, *J* = 7.0 Hz), 3.89 (q, 2H, CH_2_, *J* = 7.0 Hz), 7.19–7.35 (m, 14H, ArH), 7.74 (d, 1H, *J* = 12 Hz), 8.12 (d, 1H, *J* = 12 Hz), 10.62 (s, 1H, NH); MS *m*/*z* (%): 490 (M^+^, 4.1%), 368 (99.9%), 255 (19.9%), 105 (35.9%). Anal Calcd. for C_29_H_22_N_4_O_2_S (490.58): C, 71.00; H, 4.52; N, 11.42. Found: C, 71.12; H, 4.48; N, 11.35%.

*Ethyl 5-(3-(4-chlorophenylamino)acryloyl)-4-phenyl-2-(phenylamino)thiophene-3-carboxylate* (**9**). Yield (92%), yellow powder, m.p. 222–225 °C; IR (KBr) *v* 3426 (NH), 1652, 1622 (2 C=O) cm^−1^; ^1^H-NMR (CDCl_3_, 300 MHz) δ 0.73 (t, 3H, CH_3_, *J* = 7.0 Hz), 3.90 (q, 2H, CH_2_, *J* = 7.0 Hz), 4.63 (d, 1H, CH, *J* = 8 Hz), 6.82–7.44 (m, 15H, ArH), 10.68 (s, 1H, NH), 11.70 (s, 1H, NH); MS *m*/*z* (%) 502 (M^+^, 2.5%), 460 (46.5%), 353 (99.9%), 309 (15.5%). Anal Calcd. for C_28_H_23_ClN_2_O_3_S (503.01): C, 66.86; H, 4.61; N, 5.57. Found: C, 66.75; H, 4.48; N, 5.65%.

*Ethyl 5-(5-acetyl-6-methylpyridin-2-yl)-4-phenyl-2-(phenylamino)thiophene-3-carboxylate* (**12**). Ammonium acetate (0.156 g, 2 mmol) was added to a solution of enaminone **1** (0.42 g, 1 mmol) and 2,4-pentanedione (0.1 g, 0.1 mL, 1 mmol) in glacial acetic acid (10 mL). The reaction mixture was heated under reflux for 6 h. After cooling, the reaction mixture was poured onto ice water mixture then the formed solid product was filtered and recrystallized from ethanol to afford compound **12** as deep yellow needles, in yield (97%), m.p. 197–200 °C; IR (KBr) *v* 3424 (NH), 1708, 1675 (2C=O) cm^−1^; ^1^H-NMR (CDCl_3_, 300 MHz) δ 0.74 (t, 3H, CH_3_, *J* = 7.0 Hz), 2.44 (s, 3H, CH_3_), 2.72 (s, 3H, CH_3_), 3.92 (q, 2H, CH_2_, *J* = 7.0 Hz), 6.24 (d, 1H, *J* = 8.4 Hz), 7.11–7.46 (m, 11H, ArH), 10.54 (s, 1H, NH); MS *m*/*z* (%) 456 (M^+^, 55.1%), 259 (99.9%), 238 (27.9%), 109 (55.1%), 55 (66.2%). Anal Calcd. for C_27_H_24_N_2_O_3_S (456.56): C, 71.03; H, 5.30; N, 6.14. Found: C, 71.15; H, 5.21; N, 6.22%.

*Ethyl 5-(3-acetyl-1-phenyl-1H-pyrazole-4-carbonyl)-4-phenyl-2-(phenylamino)thiophene-3-carboxylate* (**16**). Triethylamine (2 mmol) was added to a mixture of enaminone **1** (0.42 g, 1 mmol) and the hydrazonoyl chloride **14** in dry benzene (20 mL). The resulting reaction mixture was heated under reflux for 8 h then left to cool. The solvent was distilled under reduced pressure. The residue was triturated with methanol and the solid product was collected by filtration, washed with methanol and recrystallized from ethanol to afford the pyrazole derivative **16** as pale brown powder, in yield (89%), m.p. 205–206 °C; IR(KBr) *v* 3445 (NH), 1684, 1653, 1619 (3C=O) cm^−1^; ^1^H-NMR (CDCl_3_, 300 MHz) δ 0.6 (t, 3H, CH_3_, *J* = 7.3 Hz), 2.64 (s, 3H, CH_3_), 3.8 (q, 2H, CH_2_, *J* = 7.3 Hz), 6.89–7.43 (m, 15H, ArH), 7.46 (s, 1H, CH), 10.78 (s, 1H, NH); MS *m*/*z* (%) 535 (M^+^, 85.2%), 456 (99.9%), 413 (68.4%), 217 (51.1%). Anal Calcd. for C_31_H_25_N_3_O_4_S (535.61): C, 69.52; H, 4.70; N, 7.85. Found: C, 69.38; H, 4.56; N, 7.86%.

*Ethyl 5-([1,2,4]triazolo[3,4-c][1,2,4]triazine-6-carbonyl)-4-phenyl-2-(phenyl amino)thiophene-3-carboxylate* (**19**). To a cold solution of the enaminone **1** (0.42 g, 1mmol) in pyridine (10 mL) was added portion wise the diazonium salt of aminotriazole **17** (prepared by diazotizing 3-amino-1*H*-[1,2,4]triazole (0.084 g, 1 mmol) in concentrated HNO_3_ (2 mL) with a solution of sodium nitrite (0.07 g, 1 mmol) in H_2_O (2 mL). After complete addition of the diazonium salt the reaction mixture was stirred in an ice bath for 30 min then filtered off, dried, and recrystallized from EtOH to afford the title compound as deep yellow powder (92% yield), m.p. >300 °C; IR (KBr) *v* 3418 (NH), 1657, 1622 (2C=O) cm^−1^; ^1^H-NMR (CDCl_3_, 300 MHz) δ 0.71 (t, 3H, CH_3_, *J* = 7.0 Hz), 3.88 (q, 2H, CH_2_, *J* = 7.0 Hz), 7.50–7.25 (m, 10H, ArH), 8.45 (s, 1H, CH), 9.00 (s, 1H, CH), 10.61 (s, 1H, NH); MS *m*/*z* (%) 470 (M^+^, 1.8%) for C_24_H_18_N_6_O_3_S, 455 (75.3%), 227 (35.4%), 155 (99.9%), 91 (91.1%), 69 (35.2%). Anal Calcd. for C_24_H_18_N_6_O_3_S (470.50): C, 61.27; H, 3.86; N, 17.86. Found: C, 61.38; H, 3.77; N, 17.93%.

*(E)-Ethyl 4-phenyl-2-(phenylamino)-5-(4,4,4-triethoxybut-2-enoyl)thiophene-3-carboxylate* (**21**). A mixture of enaminone derivative **1** (0.42 g, 1 mmol) and triethylorthoformate (2 mL) was heated under reflux for 2 h. After cooling, ethanol was added and the formed solid product was filtered off and recrystallized from ethanol to afford pale red needles (86% yield), m.p. 204–206 °C; IR (KBr) *v* 3462 (NH), 1658, 1619 (2C=O), 1554 (C=C) cm^−1^; (DMSO-*d*_6_, 400 MHz) δ 0.66 (t, 3H, CH_3_, *J =* 7.3 Hz), 1.24 (t, 9H, 3CH_3_, *J =* 7.3 Hz), 3.3 (q, 6H, 3CH_2_, *J* = 7.3 Hz), 3.85 (q, 2H, CH_2_, *J* = 7.3 Hz), 4.31 (d, 1H, CH, *J* = 12.4 Hz, CH^α^=CH^β^), 5.11 (d, 1H, CH, *J* = 12.4 Hz, CH^α^=CH^β^), 7.2–7.4 (m, 10H, ArH), 10.28 (s, 1H, NH); ^13^C-NMR (DMSO-*d*_6_, 100 MHz;) 179.9, 162.4, 157.8, 149.7, 147.3, 140.4, 135.9, 133.0, 128.4, 127.9, 127.8, 126.5, 126.1, 120.8, 118.4, 117.8, 92.6, 58.9, 53.4, 22.7, 12.2; MS *m*/*z* (%) 523 (M^+^, 1.3%), 511 (16%), 270 (40%), 180 (29.8%), 91 (99.9%). Anal Calcd. for C_29_H_33_NO_6_S (523.64): C, 66.52; H, 6.35; N, 2.67. Found: C, 66.48; H, 6.42; N, 2.55%.

### 3.2. Agar Diffusion Well Method to Determine the Antimicrobial Activity

The microorganism inoculums were uniformly spread using sterile cotton swabs on a sterile Petri dish malt extract agar (for fungi) and nutrient agar (for bacteria). One hundred microliters of each sample were added to each well (10 mm diameter holes cut in the agar gel, 20 mm apart from one another). Then, the systems were incubated for 24–48 h at 37 °C (for bacteria) and at 28 °C (for fungi). After incubation, the microorganism’s growth was observed. Inhibition of the bacterial and fungal growth were measured in mm. Tests were performed in triplicate [31].

## 4. Conclusions

In conclusion various thiophene derivatives were synthesized in efficient and easy protocol. Antimicrobial studies revealed that compounds **9**, **12**, and **19** showed a higher activity towards *Aspergillus fumigates* than the standard drug Amphotericin B.

## Figures and Tables

**Table 1:** Antibacterial activity of the synthesized compounds.

Sample	Gram Positive Bacteria		Gram Negative Bacteria	
	*Streptococcus pneumoniae*	*Bacillis subtilis*	*Pseudomonas aeruginosa*	*Escherichia coli*
	Ampicillin		Gentamicin	
**St**	23.8 ± 0.2	32.4 ± 0.3	17.3 ± 0.1	19.9 ± 0.3
**4**	12.3 ± 0.58	12.7 ± 0.37	9.8 ± 0.34	11.3 ± 0.39
**7**	14.6 ± 0.58	14.3 ± 0.58	10.2 ± 0.31	8.8 ± 0.24
**9**	13.8 ± 0.40	17.2 ± 0.43	11.6 ± 0.36	10.9 ± 0.21
**12**	12.9 ± 0.63	13.2 ± 0.58	10.7 ± 0.24	9.9 ± 0.34
**16**	17.5 ± 0.44	19.8 ± 0.63	15.1 ± 0.45	18.0 ± 0.25
**19**	13.8 ± 0.4	17.2 ± 0.43	11.6 ± 0.36	10.9 ± 0.21

**Table 2:** Antifungal activity of the synthesized compounds.

Sample	Fungi			
	*Aspergillus fumigates*	*Syncephalastrum racemosum*	*Geotricum candidum*	*Candida albicans*
	Amphotericin B			
**St**	23.7 ± 0.1	19.7 ± 0.2	28.7 ± 0.2	25.4 ± 0.1
**4**	15.7 ± 0.47	16.6 ± 0.62	12.6 ± 0.38	12.7 ± 0.37
**7**	16.3 ± 0.35	14.8 ± 0.46	15.3 ± 0.52	14.3 ± 0.58
**9**	24.1 ± 0.51	16.9 ± 0.52	11.5 ± 0.43	10.8 ± 0.46
**12**	28.1 ± 0.76	23.4 ± 0.77	14.1 ± 0.65	13.2 ± 0.58
**16**	15.3 ± 0.55	13.4 ± 0.35	11.5 ± 0.58	13.1 ± 0.3
**19**	24.1 ± 0.51	16.9 ± 0.52	11.5 ± 0.34	10.8 ± 0.46
